# Supplementary material for: Development of Protein- and Fiber-Enriched, Sugar-Free Lentil Cookies: Impact of Whey Protein, Inulin, and Xylitol on Physical, Textural, and Sensory Characteristics
Source: Foods. 2022 Nov 26;11(23):3819. doi: 10.3390/foods11233819 (PMC9736369; doi:10.3390/foods11233819)
Supplement: Supplementary file 1 [file foods-11-03819-s001.zip › foods-2050479-Supplementary materials.pdf]

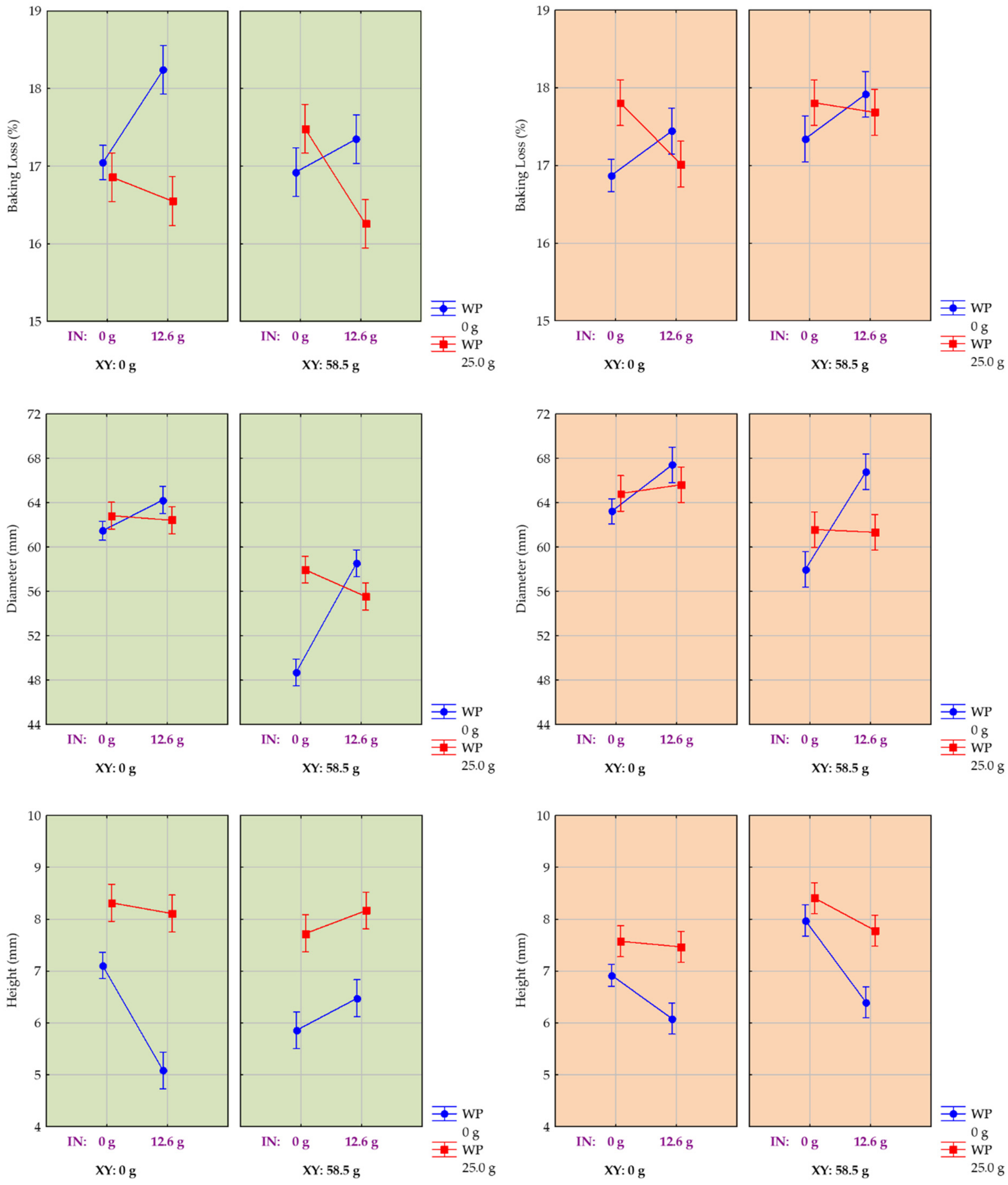

(**a**) (**b**)

Figure S1. Interaction plots for the baking loss, diameter and height of (a) green-lentil- and (b) red-lentil-based cookies. Data markers represent means. Vertical bars denote 95% confidence intervals (n=12). XY: xylitol, IN: inulin, WP: whey protein isolate.

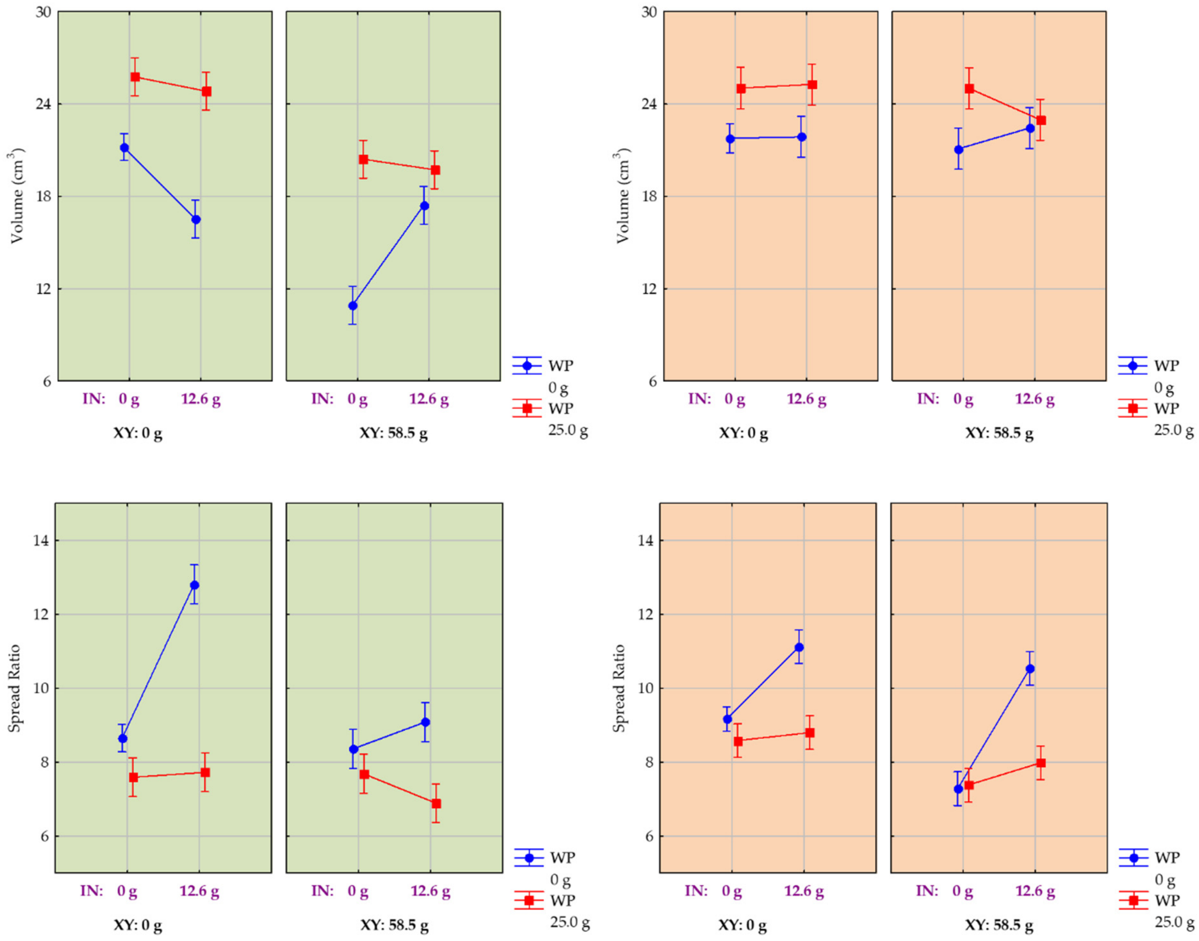

(**a**) (**b**)

Figure S2. Interaction plots for the volume and spread ratio of (a) green-lentil- and (b) red-lentil-based cookies. Data markers represent means. Vertical bars denote 95% confidence intervals (n=12). XY: xylitol, IN: inulin, WP: whey protein isolate.

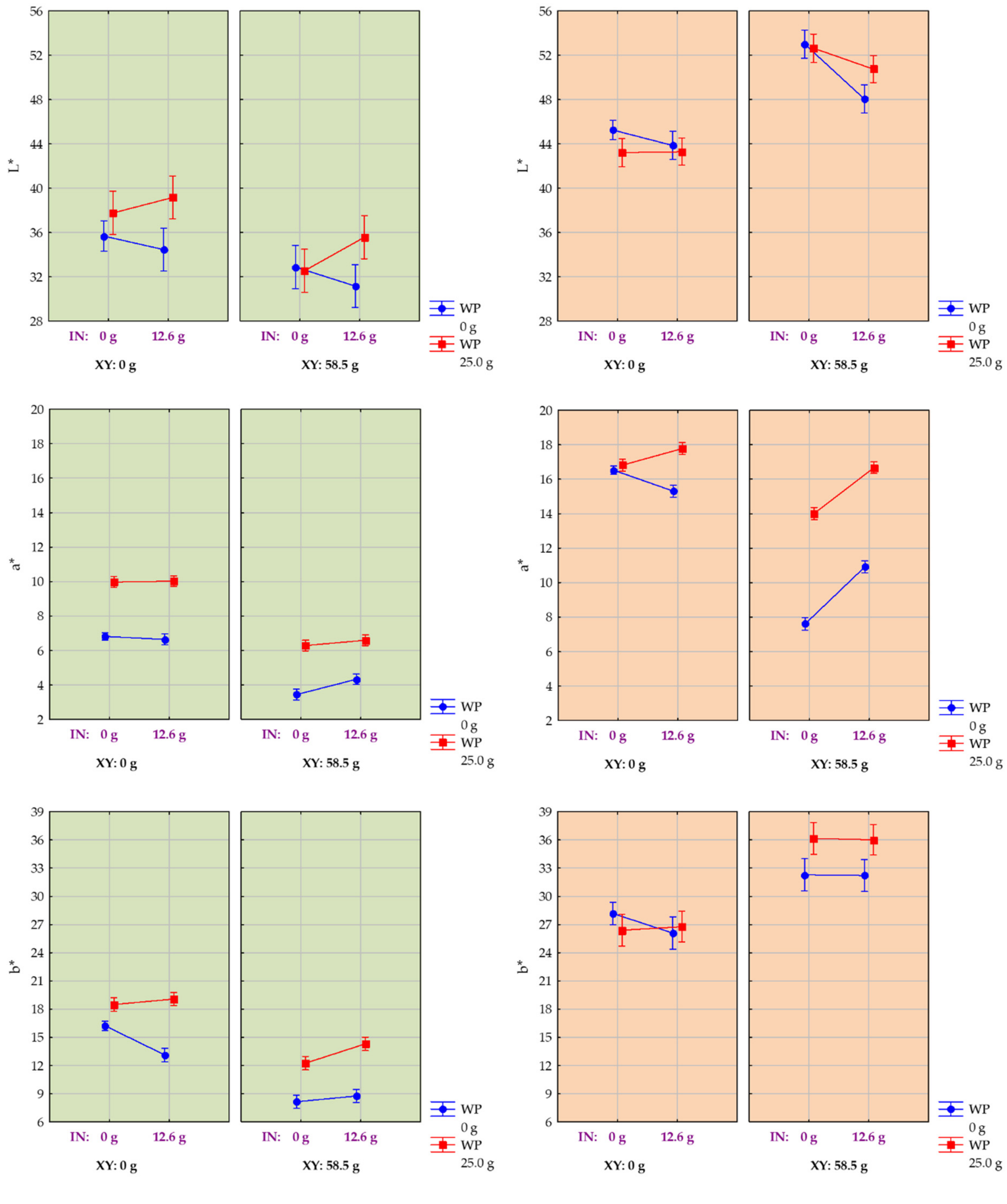

(**a**) (**b**)

Figure S3. Interaction plots for the color parameters of (a) green-lentil- and (b) red-lentil-based cookies. Data markers represent means. Vertical bars denote 95% confidence intervals (n=12). XY: xylitol, IN: inulin, WP: whey protein isolate.

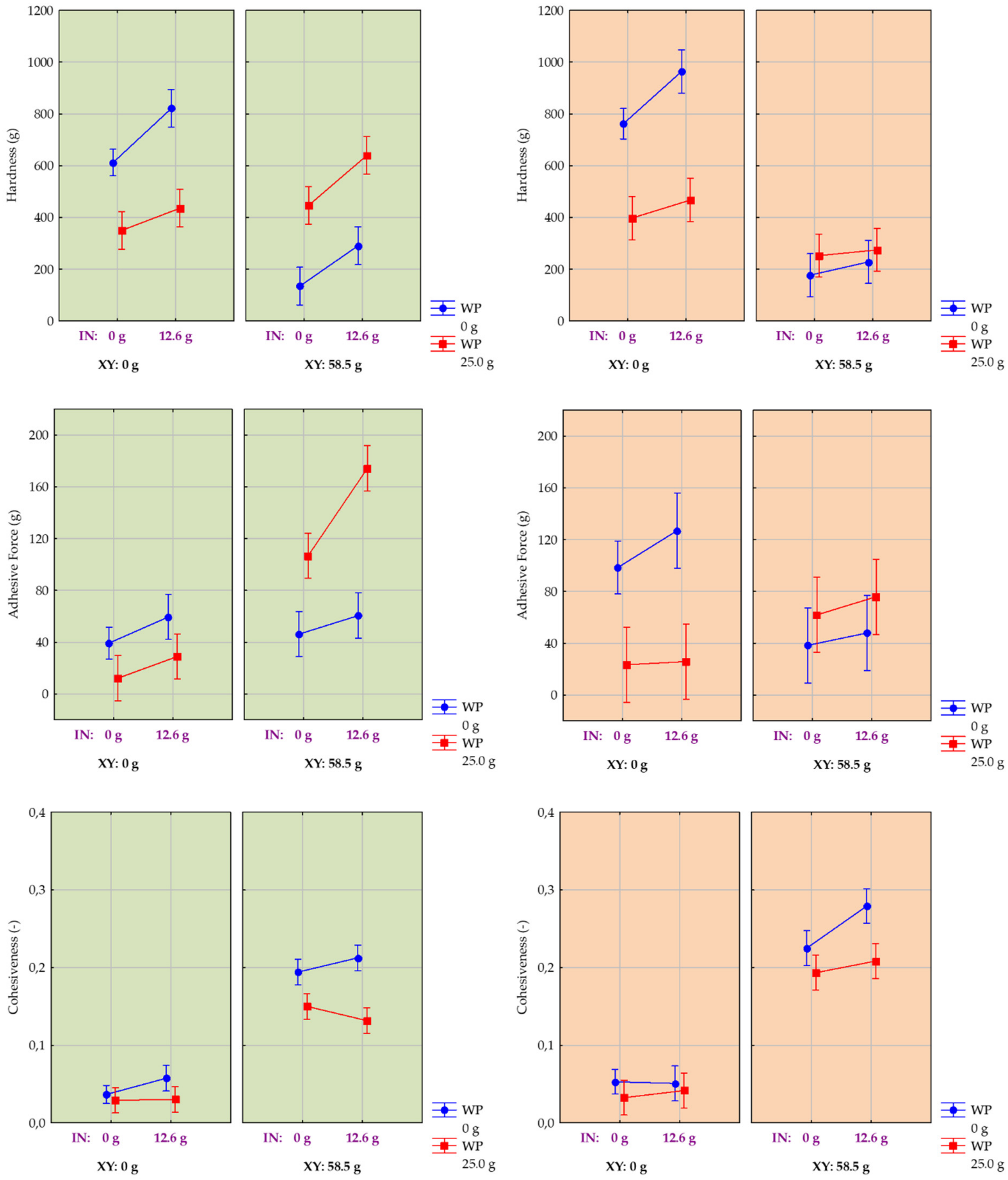

(**a**) (**b**)

Figure S4. Interaction plots for the textural properties of (a) green-lentil- and (b) red-lentil-based cookies. Data markers represent means. Vertical bars denote 95% confidence intervals (n=12). XY: xylitol, IN: inulin, WP: whey protein isolate.

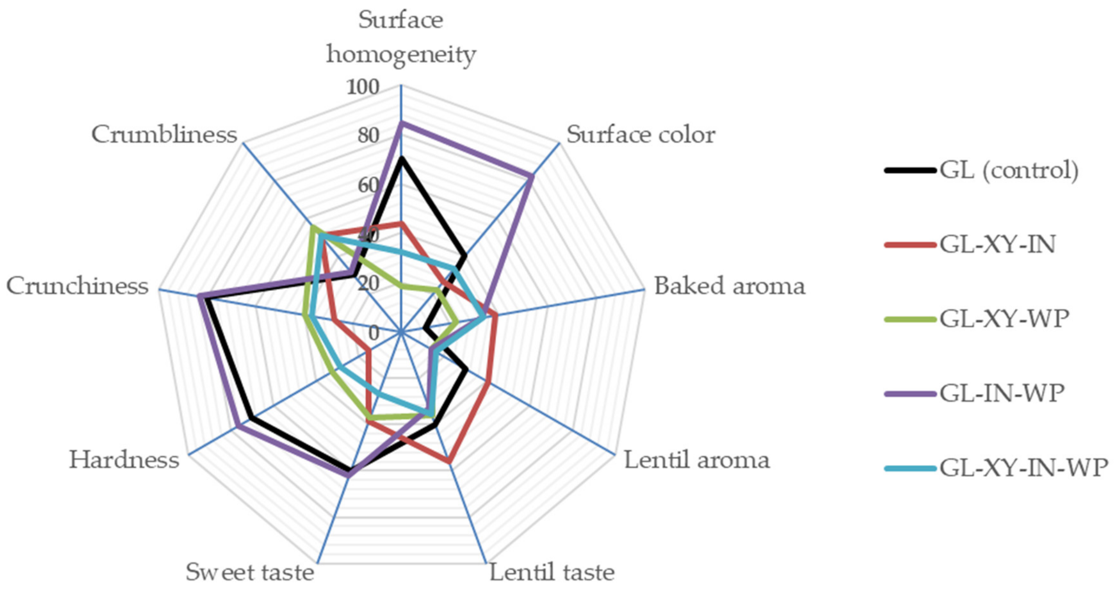

Figure S5. Sensory profile of green-lentil-based cookies. The values are the average from thirteen panelists. GL: green lentil, XY: xylitol, IN: inulin, WP: whey protein isolate.

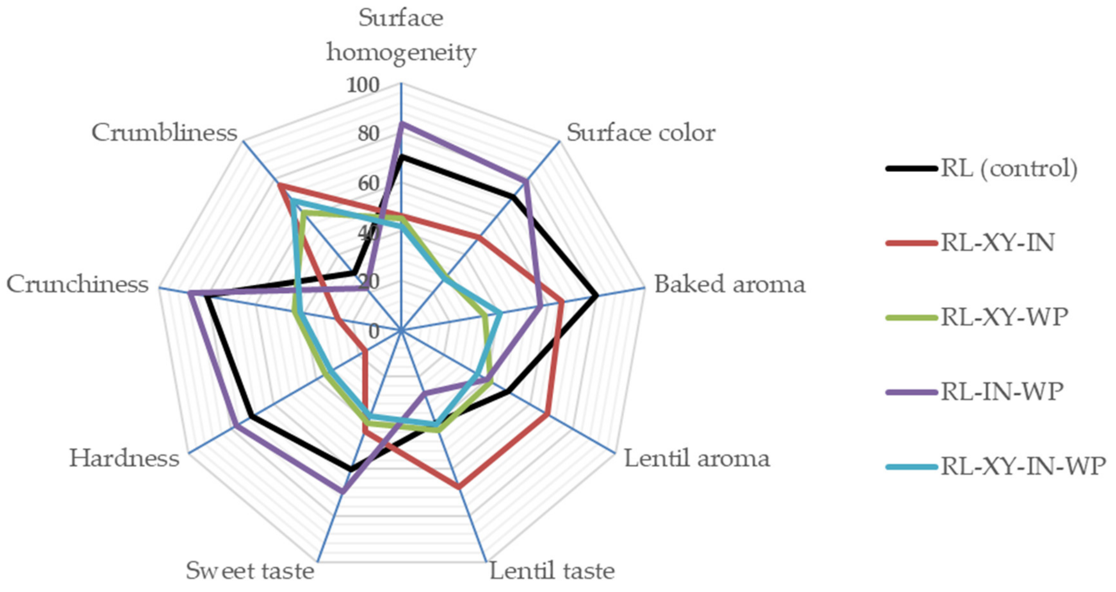

Figure S6. Sensory profile of red-lentil-based cookies. The values are the average from sixteen panelists. RL: red lentil, XY: xylitol, IN: inulin, WP: whey protein isolate.
